# Monitoring of species’ genetic diversity in Europe varies greatly and overlooks potential climate change impacts

**DOI:** 10.1038/s41559-023-02260-0

**Published:** 2024-01-15

**Authors:** Peter B. Pearman, Olivier Broennimann, Tsipe Aavik, Tamer Albayrak, Paulo C. Alves, F. A. Aravanopoulos, Laura D. Bertola, Aleksandra Biedrzycka, Elena Buzan, Vlatka Cubric-Curik, Mihajla Djan, Ancuta Fedorca, Angela P. Fuentes-Pardo, Barbara Fussi, José A. Godoy, Felix Gugerli, Sean Hoban, Rolf Holderegger, Christina Hvilsom, Laura Iacolina, Belma Kalamujic Stroil, Peter Klinga, Maciej K. Konopiński, Alexander Kopatz, Linda Laikre, Margarida Lopes-Fernandes, Barry John McMahon, Joachim Mergeay, Charalambos Neophytou, Snæbjörn Pálsson, Ivan Paz-Vinas, Diana Posledovich, Craig R. Primmer, Joost A. M. Raeymaekers, Baruch Rinkevich, Barbora Rolečková, Dainis Ruņģis, Laura Schuerz, Gernot Segelbacher, Katja Kavčič Sonnenschein, Milomir Stefanovic, Henrik Thurfjell, Sabrina Träger, Ivaylo N. Tsvetkov, Nevena Velickovic, Philippine Vergeer, Cristiano Vernesi, Carles Vilà, Marjana Westergren, Frank E. Zachos, Antoine Guisan, Michael Bruford

**Affiliations:** 1 Department of Plant Biology and Ecology, Faculty of Sciences and Technology, University of the Basque Country UPV/EHU, Leioa, Spain; 2 IKERBASQUE Basque Foundation for Science, Bilbao, Spain; 3 BC3 Basque Center for Climate Change, Leioa, Spain; 4 Department of Ecology and Evolution, Biophore, University of Lausanne, Lausanne, Switzerland; 5 Institute of Earth Surface Dynamics, Geopolis, University of Lausanne, Lausanne, Switzerland; 6 Institute of Ecology and Earth Sciences, University of Tartu, Tartu, Estonia; 7 Science and Art Faculty, Department of Biology, Lab of Ornithology, Burdur Mehmet Akif Ersoy University, Burdur, Turkey; 8 CIBIO-InBIO Laboratório Associado & Departamento de Biologia, Faculdade de Ciências do Porto, Campus de Vairão, Universidade do Porto, Vairão, Portugal; 9 BIOPOLIS Program in Genomics, Biodiversity and Land Planning, CIBIO, Campus de Vairão, Universidade do Porto, Vairão, Portugal; 10 EBM, Estação Biológica de Mértola, Mértola, Portugal; 11 Faculty of Agriculture, Forest Science and Natural Environment, Aristotle University of Thessaloniki, Thessaloniki, Greece; 12 Department of Biology, University of Copenhagen, Copenhagen, Denmark; 13 Institute of Nature Conservation, Polish Academy of Sciences, Kraków, Poland; 14 Faculty of Mathematics, Natural Sciences, and Information Technologies, University of Primorska, Koper, Slovenia; 15 Faculty of Environmental Protection, Velenje, Slovenia; 16 Department of Animal Science, University of Zagreb, Zagreb, Croatia; 17 Department of Biology and Ecology, Faculty of Sciences, University of Novi Sad, Novi Sad, Serbia; 18 Department of Wildlife, National Institute for Research and Development in Forestry ‘Marin Dracea’, Brasov, Romania; 19 Department of Silviculture, Faculty of Silviculture and Forest Engineering, Transilvania University of Brasov, Brasov, Romania; 20 Department of Medical Biochemistry and Microbiology, Uppsala University, Uppsala, Sweden; 21 Bavarian Office for Forest Genetics, Teisendorf, Germany; 22 Doñana Biological Station (EBD-CSIC), Seville, Spain; 23 Swiss Federal Research Institute WSL, Birmensdorf, Switzerland; 24 Center for Tree Science, Morton Arboretum, Lisle, IL USA; 25 Department of Environmental Systems Sciences D-USYS, ETH Zürich, Zürich, Switzerland; 26 Copenhagen Zoo, Frederiksberg, Denmark; 27 Faculty of Mathematics, Natural Sciences and Information Technologies, Department of Biodiversity, University of Primorska, Koper, Slovenia; 28 Department of Veterinary Medicine, University of Sassari, Sassari, Italy; 29 Institute for Genetic Engineering and Biotechnology, University of Sarajevo, Sarajevo, Bosnia and Herzegovina; 30 Faculty of Forestry, Technical University in Zvolen, Zvolen, Slovak Republic; 31 Department of Forest Ecology, Faculty of Forestry and Wood Sciences, Czech University of Life Sciences, Prague, Czech Republic; 32 Norwegian Institute for Nature Research, Trondheim, Norway; 33 Department of Zoology, Division of Population Genetics, Stockholm University, Stockholm, Sweden; 34 Centre for Research in Anthropology, Lisbon, Portugal; 35 Institute for Nature Conservation and Forests, Lisbon, Portugal; 36 UCD School of Agriculture and Food Science, University College Dublin, Dublin, Ireland; 37 Research Institute for Nature and Forest, Geraardsbergen, Belgium; 38 Ecology, Evolution and Biodiversity Conservation, KU Leuven, Leuven, Belgium; 39 Institute of Silviculture, Department of Forest and Soil Sciences, University of Natural Resources and Life Sciences (BOKU), Vienna, Austria; 40 Department of Forest Nature Conservation, Forest Research Institute Baden-Württemberg, Freiburg, Germany; 41 Department of Biology, University of Iceland, Reykjavik, Iceland; 42 Department of Biology, Colorado State University, Fort Collins, CO USA; 43 Faculty of Biological & Environmental Sciences, University of Helsinki, Helsinki, Finland; 44 Faculty of Biosciences and Aquaculture, Nord University, Bodø, Norway; 45 Israel Oceanographic and Limnological Research, National Institute of Oceanography, Haifa, Israel; 46 Institute of Vertebrate Biology, Czech Academy of Sciences, Brno, Czech Republic; 47 Genetic Resource Centre, Latvian State Forest Research Institute ‘Silava’, Salaspils, Latvia; 48 Wildlife Ecology and Management, University Freiburg, Freiburg, Germany; 49 Slovenian Forestry Institute, Ljubljana, Slovenia; 50 Swedish Species Information Centre, Swedish University of Agricultural Sciences, Uppsala, Sweden; 51 Institute of Biology/Geobotany and Botanical Garden, Martin Luther University Halle-Wittenberg, Halle (Saale), Germany; 52 German Centre for Integrative Biodiversity Research (iDiv) Halle-Jena-Leipzig, Leipzig, Germany; 53 Department of Forest Genetics, Physiology and Plantations, Forest Research Institute, Bulgarian Academy of Sciences, Sofia, Bulgaria; 54 Plant Ecology and Nature Conservation Group, Wageningen University, Wageningen, the Netherlands; 55 Forest Ecology Unit, Research and Innovation Centre, Fondazione Edmund Mach, San Michele all’Adige, Italy; 56 Natural History Museum Vienna, Vienna, Austria; 57 Department of Evolutionary Biology, University of Vienna, Vienna, Austria; 58 Department of Genetics, University of the Free State, Bloemfontein, South Africa; 59 School of Biosciences, Cardiff University, Cardiff, UK; 60 Department of Biochemistry, Genetics and Molecular Biology, University of Pretoria, Pretoria, South Africa

**Keywords:** Conservation biology, Climate-change ecology, Climate-change impacts, Genetic variation, Biodiversity

## Abstract

Genetic monitoring of populations currently attracts interest in the context of the Convention on Biological Diversity but needs long-term planning and investments. However, genetic diversity has been largely neglected in biodiversity monitoring, and when addressed, it is treated separately, detached from other conservation issues, such as habitat alteration due to climate change. We report an accounting of efforts to monitor population genetic diversity in Europe (genetic monitoring effort, GME), the evaluation of which can help guide future capacity building and collaboration towards areas most in need of expanded monitoring. Overlaying GME with areas where the ranges of selected species of conservation interest approach current and future climate niche limits helps identify whether GME coincides with anticipated climate change effects on biodiversity. Our analysis suggests that country area, financial resources and conservation policy influence GME, high values of which only partially match species’ joint patterns of limits to suitable climatic conditions. Populations at trailing climatic niche margins probably hold genetic diversity that is important for adaptation to changing climate. Our results illuminate the need in Europe for expanded investment in genetic monitoring across climate gradients occupied by focal species, a need arguably greatest in southeastern European countries. This need could be met in part by expanding the European Union’s Birds and Habitats Directives to fully address the conservation and monitoring of genetic diversity.

## Abstract (web summary)

Comparing data on genetic monitoring efforts across Europe with the distributions of areas at species’ climatic niche margins, the authors show that monitoring efforts should be expanded to populations at trailing niche margins to include genetic variation that may prove important for adaptation to ongoing climate warming.

## Main

The maintenance of wild population genetic diversity (PGD) is an important component of the Convention on Biological Diversity (CBD)^1^, but it has received little international attention until recently^1–4^, limiting our ability to monitor and manage wild populations to sustain PGD^5^. The resulting urgent need for expanded monitoring of PGD motivates the development of globally implementable indicators of genetic diversity^6–9^, some of which are included in the recently adopted CBD Kunming-Montreal Global Biodiversity Framework^3,10^. But while ongoing anthropogenic loss of PGD is being documented^11–13^, efforts to detect climate change effects on PGD are taxonomically and geographically limited^14,15^ and are absent from international biodiversity agreements. Populations in extreme climatic conditions, such as those near trailing climatic niche margins, are particularly relevant to species’ potential for adaptation to a changing climate^16^. Nonetheless, multispecies patterns of populations near trailing niche margins, which can serve as potential indicators of areas important for the adaptive potential of multiple species and thus reveal possible PGD monitoring sites, remain unidentified. This suggests the need for improved quantification of the relationships between species’ niche limits along environmental gradients and associated PGD^17,18^.

Species populations close to their environmental niche margins may differ genetically from those at the niche centre and influence the course of adaptation to changing environments^19,20^. Evidence shows that populations at niche margins towards stressful environmental extremes are locally adapted^21^, having distinguishable genetic architecture independent of their geographic position within the species range^22^. Populations near trailing niche limits probably hold important, adaptive genetic variants^22–24^ that can reduce predicted range loss^18,25^ and contribute to the adaptation of environmentally central populations^26^ to a warming, drying climate, despite greater gene flow from the niche centre to these marginal populations^27^. But genetic diversity and adaptive variants held in marginal populations may be lost (1) when gene flow to environmentally central areas is impeded, (2) when genetic drift strongly affects populations with small effective population sizes or (3) if the populations go extinct as climate extremes eventually exceed species’ tolerances^28^. These results suggest that global genetic monitoring frameworks^10^ need to anticipate climate impacts, collect samples across entire climate gradients and evaluate the contributions of marginal populations to genetic diversity and adaptive potential^29^. However, no previous accounting of recent and historical PGD monitoring exists, leaving us ignorant of taxonomic, national and geographic trends in monitoring effort, and hampering our capacity to detect changing PGD and adaptive potential under climate change threat. Yet, even without such accounting, existing PGD monitoring efforts suggest notable resources, infrastructure and political support, and can serve as an index of current and potential future genetic monitoring effort (GME).

Here we examine the gap between GME and the need for genetic monitoring generated by deteriorating climatic conditions by asking the following questions. (1) How is GME distributed across Europe, and on which taxa has PGD monitoring focused? (2) Which factors explain among-country variation in GME? (3) How will countries differ in the exposure of threatened species to climate change? Finally, (4) how does GME coincide with anticipated impacts of climate change on habitat suitability for populations? Using evidence of monitoring from the peer-reviewed and technical literature, we examine how 38 countries in the European Commission’s Cooperation in Science and Technology (COST) programme^30^ demonstrate GME for purposes of biodiversity conservation and management. The collective use of COST full-member countries as a study area allowed us to cover much of the European continent and major islands. We explain variation in GME in these countries in relation to two fundamental national characteristics: per capita gross domestic product (GDP) and area. One could also expect greater GME in southern Europe in recognition of greater habitat diversity, species endemicity and biodiversity hotspots than in the north^31,32^.

We used climate and biological data to stratify species ranges into areas with core climatic niche conditions and areas with conditions near niche limits (that is, areas of niche marginality), distinguishing areas with trailing niche margins due to climate change. We then compared a multispecies indicator of trailing niche marginality to country GME, to directly relate GME to climate-driven decline in niche conditions for multiple species. To do this, we estimated and mapped the range-wide predicted impacts of climate change on the present and future geographic distributions of climatic niche marginality^33^. We did this for species in four groups, selected for recognized and potential conservation and management interest (amphibians, large birds, carnivorans and forest trees). Within COST member countries, we aggregated the climate change impacts on these groups of species by tallying a count of niche marginal species, thereby defining the pattern of trailing climate niche marginality among countries. Finally, we plotted this indicator of cumulative climate impacts on species against values of country GME.

## Results

**Extended Data Fig. 1:**
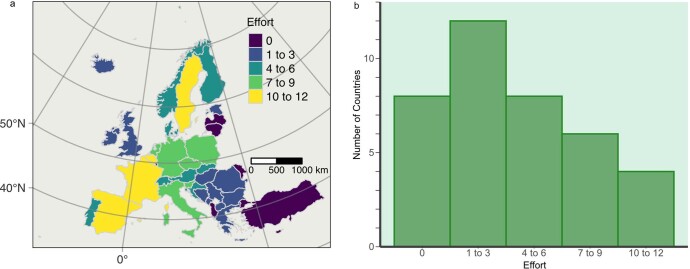
Documented programs to monitor population genetic diversity for conservation and management in COST member countries, as an indicator of genetic monitoring effort, GME, up to 31 December 2021. (**a**) The geographic distribution of monitoring effort to countries, as a tally across all domestic and wild terrestrial and marine species indicates that countries with relatively high effort for monitoring are found in both northern and southern Europe. COST countries in southeastern Europe present generally low genetic monitoring effort. (**b**) The distribution of programs to countries shows that most countries have established six or fewer monitoring programs.

Between 22 November 2019 and 31 December 2021, we received 480 submissions of candidate monitoring projects from conservation geneticists, practitioners and stakeholders. These submissions responded to a variety of data fields that described candidate projects (Table 1). We evaluated these for validity as Category II genetic monitoring projects^34^, which report temporally separate assessments of PGD metrics of one or more populations of a species. We focus here exclusively on this type of genetic monitoring because it directly tracks PGD over time, while we recognize that other types of genetic monitoring, including genetic assessments and species identification programmes, are also highly relevant to conservation but address questions other than the change in PGD over time. We found 38 additional candidate Category II monitoring projects through a structured search of the Web of Science. Of the total 518 candidates, we identified 103 as valid Category II monitoring projects, the vast majority of which report sampled populations from one (84) or two (14) countries^35^. We tallied international and transboundary projects separately by country, and we documented a total of 151 national-level projects of Category II genetic monitoring. We found Category II monitoring in 30 of 38 COST countries that were full members at the beginning of data solicitation (Extended Data Fig. 1a,b).

**Table 1:** Requested information to characterize submitted monitoring projects/programmes

Variable	Values
Contributor	First and last name(s)
Description of project	Text description provided by contributor
Programme/project name	Text name, not available
Barcoding study	True/false
Within-species diversity	True/false
Temporal category	‘Snapshot’, ‘Horizontal’
Annual sampling?	True/false
Country	One or more country names
Political extent	Regional, national, multi-national
Marker type	Organelle sequence, other autosomal, SNP, microsatellite, sex chromosome, multi-marker
Strict/relaxed	‘Strict’ indicates study was a priori designed as a monitoring study; ‘relaxed’ if data used post-hoc for monitoring
Focal groups (true/false)	Carnivora, bear, wolf, lynx, other mammal, Aves, Insecta, fish, marine, plant, forest trees, amphibians, other, domesticated/captive
Name(s) of focal taxon/taxa	Common names (English), scientific names
EU Directive (Habitats or Birds) and Annex	EU Directive and Annex listing for each monitored species
Documentation/document type	Project report in national language, project report in English, government report in national language, other report in national language, scientific publication, not available
Document format	PDF, link, paper copy, not available
Document locator	DOI if available
Document title or reference	Complete citation when available
Project or report webpage	URL listed when available
Notes	Unrestricted text

### GME

**Fig. 1:**
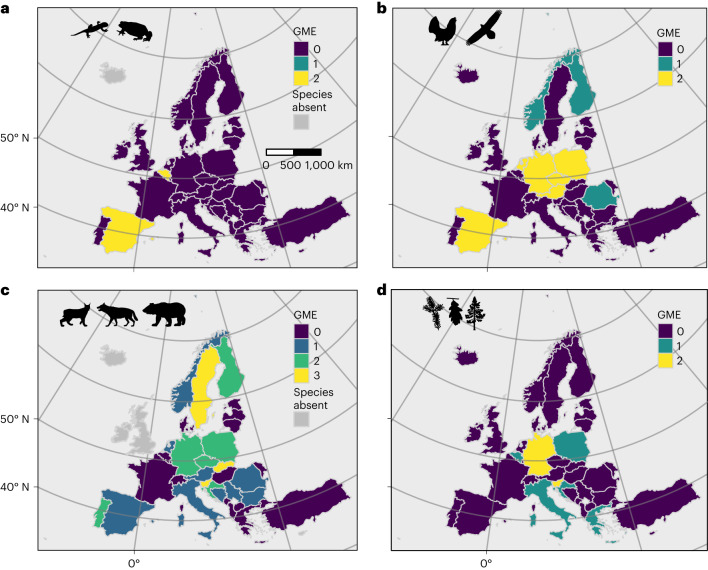
Geographic distribution of effort to monitor population genetic diversity (GME), for purposes of conservation or management, among COST full-member countries. **a**–**d**, The tally of genetic monitoring programmes for amphibians (**a**), birds (**b**), carnivorans (**c**) and forest trees (**d**). The programmes included here are consistent with the requirements for Category II monitoring, and they offer documentation of multiple estimates over time of at least one index of genetic diversity. Few countries have GME for amphibians, while most countries have established at least one programme for a carnivoran species. Source data

**Extended Data Fig. 2:**
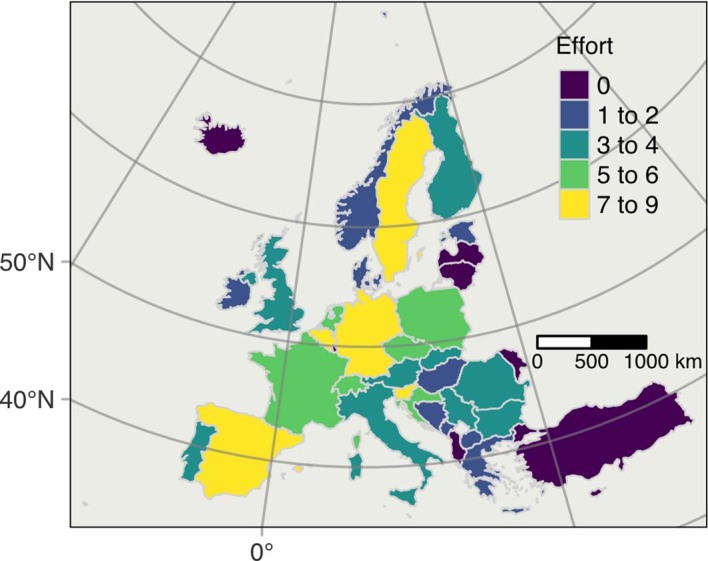
Genetic monitoring effort, GME, for terrestrial species. The map shows for each COST Full-Member country the number of the monitoring projects up to 31 December 2021, The data include projects monitoring amphibians, birds, insects, carnivorans, other mammals and trees, but exclude programs/projects that monitored fish, marine species and domesticated/captive species.

**Extended Data Fig. 3:**
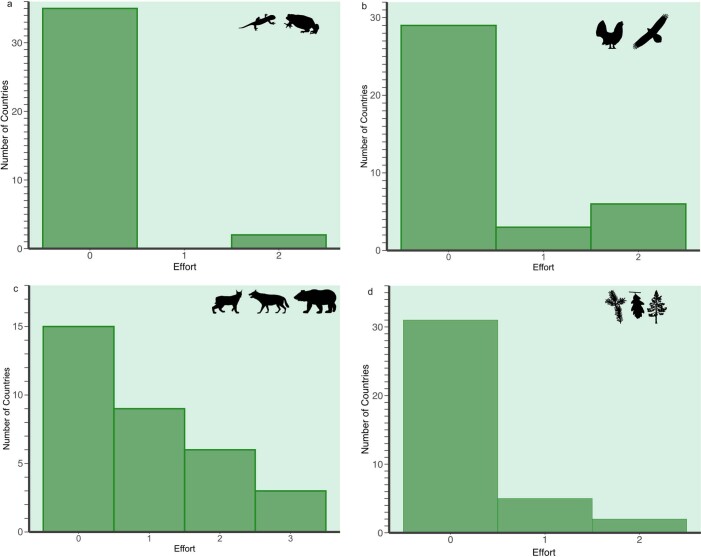
Frequency distribution of genetic monitoring effort among COST Full-Member Countries. The data are for (**a**) amphibians, (**b**) birds, **(c)** large European carnivorans, and (**d**) forest trees. They represent all projects and programs using genetic data and reporting data on genetic diversity, from at least two time points, thus qualifying as Category II monitoring of population genetic diversity. Attribution for all silhouettes in this paper: https://www.phylopic.org/permalinks/ec1bdb9ee275ab1dc5a68e090a169a96c06c559e516989215bb5d881696db666.

**Extended Data Fig. 4:**
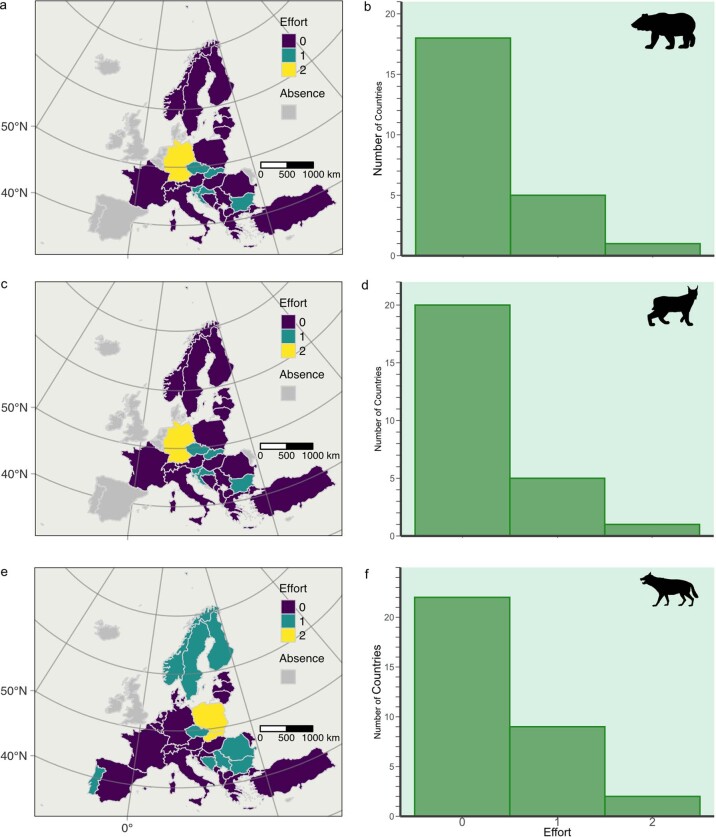
Distribution and frequency of monitoring programs for three carnivorans in COST Full-Member Countries. The data are for (**a, b**) Eurasian brown bear,*Ursus arctos*, (**c, d**) Eurasian lynx, *Lynx lynx*, and (**e, f**) Eurasian wolf, *Canis lupus*. Tallies are of identifiably distinct monitoring projects and programs that report Category II monitoring of population genetic diversity.

We documented a maximum of 12 projects for Belgium and Sweden and 11 projects for Spain and France (Extended Data Fig. 1a). We found no GME in eight countries (Extended Data Fig. 1b), including ones as geographically and economically disparate as Turkey and Luxemburg. This pattern is robust to the exclusive consideration of terrestrial wild species (that is, the exclusion of programmes monitoring fish, marine species and domesticated/captive populations; Extended Data Fig. 2). The GME of COST countries varies greatly by taxonomic and functional groups. For example, while many amphibians are of recognized conservation concern, only two European countries demonstrated GME for amphibians (Belgium and Spain; Fig. 1a). Many more countries (nine) have monitored PGD in at least one bird species (Fig. 1b and Extended Data Fig. 3b). Approximately half of COST countries (18) have monitored PGD in one or more large carnivorans (Fig. 1c and Extended Data Fig. 3c), although certain carnivorans are absent from some COST countries (Extended Data Fig. 4a,c,e). In contrast, while all COST countries have tree species, less than one quarter of COST countries (seven) have monitored PGD in at least one of these species (Fig. 1d and Extended Data Fig. 3d). Additional monitoring effort has focused on fish, marine species and insects (Appendix 1, Supplementary Information; all supplementary materials are available at 10.5281/zenodo.8417583).

**Fig. 2:**
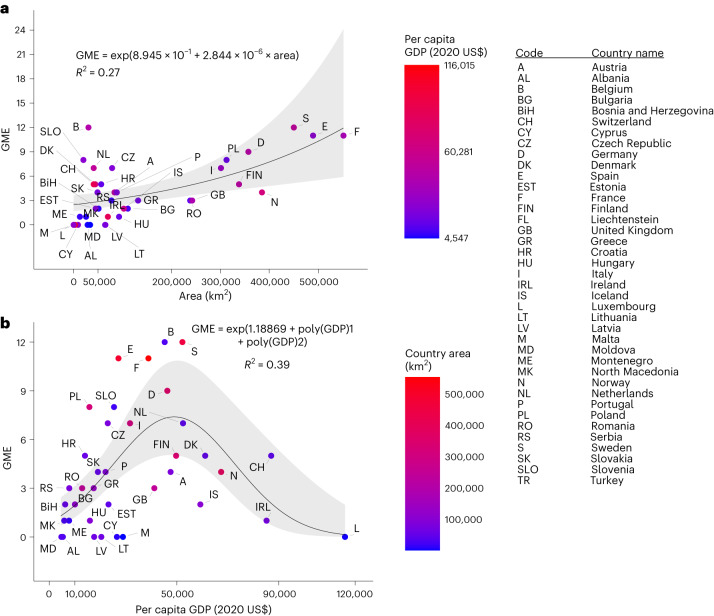
GME of COST full-member countries as a function of area per capita GDP. **a**,**b**, Generalized linear models for the GME of COST full-member countries, represented by international postal codes, as a function of area (**a**) and per capita GDP (**b**). The equations of the lines are shown, along with 95% confidence intervals in shading. The models were fit as negative binomial distributions with the log link function. Model fit is given as Veall-Zimmermann *R*^2^. Turkey is of substantially greater geographic extent than the displayed countries, but it has no documented GME and is omitted as an outlier and influential observation. Both the linear area term and the quadratic GDP term are significant in the multiple generalized model corrected for spatial autocorrelation (two-tailed tests; area: *z* = 2.269, *P* < 0.0233; GDP quadratic: *z* = −2.969, *P* = 0.00299; see the Methods for the details). A significant quadratic term remains upon the omission of any one of the three high-GDP countries. Source data

Turkey is by far the largest COST country by area, and with almost 784,600 km^2^, it is 42% larger than the next largest country, France (excluding its overseas territories). With no documented PGD monitoring, Turkey is an outlier for its size and absence of GME and is an influential observation in statistical analysis. When Turkey is omitted, analysis of the other COST countries demonstrates that larger countries tend to have higher GME (Fig. 2a; negative binomial regression, *P* = 0.02). In contrast, intermediate GDP is associated with greater GME (Fig. 2b; negative binomial regression, GDP quadratic term *P* = 0.003; Appendix 2, Supplementary Information). Substantial residual variation remains, with Austria, Finland, Norway and the United Kingdom among those countries having fewer projects than expected, and Belgium and Sweden more projects, in relation to both size and GDP (Fig. 2b). The negative quadratic relationship of GME with GDP remains statistically significant with the omission of data from any single outlier or extreme value (Switzerland, Ireland or Luxembourg; Appendix 2, Supplementary Information).

### Joint environmental niche marginality framework

**Fig. 3:**
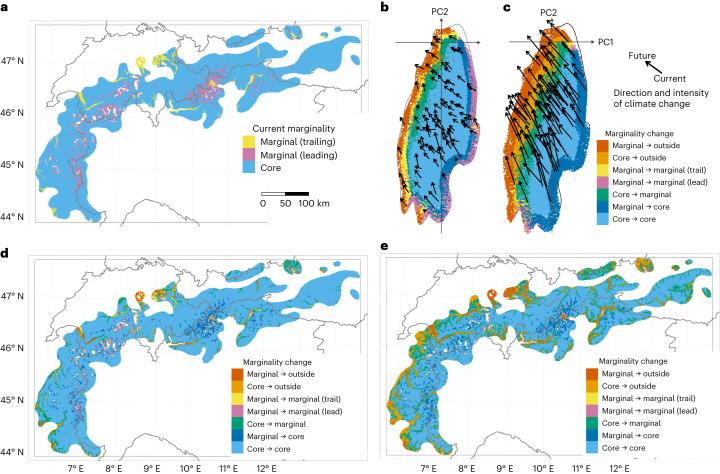
Current and future climatic niche marginality for the Swiss stone pine (*Pinus cembra*) by 2041–2070. **a**, Current marginal and core areas. Marginal areas are split into trailing and leading edges on the basis of differences between current and future Niche Margin Index (NMI) values: NMI values increase at the leading edge; NMI values decrease at the trailing edge. **b**, Predicted changes in climatic marginality according to the mildest climate change scenario (MPI-ESM1-2-HR, SSP 3-7.0). All pixels of the range of the species are represented in the principal component (PC) analysis space with coloured points corresponding to categories of possible changes in climatic marginality. The direction and intensity of change in climatic conditions for a random subset of 100 pixels are indicated with arrows. **c**, Predicted changes in climatic marginality according to the harshest climate change scenario (UKESM1-0-LL, SSP 5-8.5). **d**, Changes in climatic marginality categories across the range of the species for climate change scenario MPI-ESM1-2-HR, SSP 3-7.0 (geographic representation of **b**). **e**, Changes in climatic marginality categories across the range of the species for climate change scenario UKESM1-0-LL, SSP 5-8.5 (geographic representation of **c**).

**Extended Data Fig. 5:**
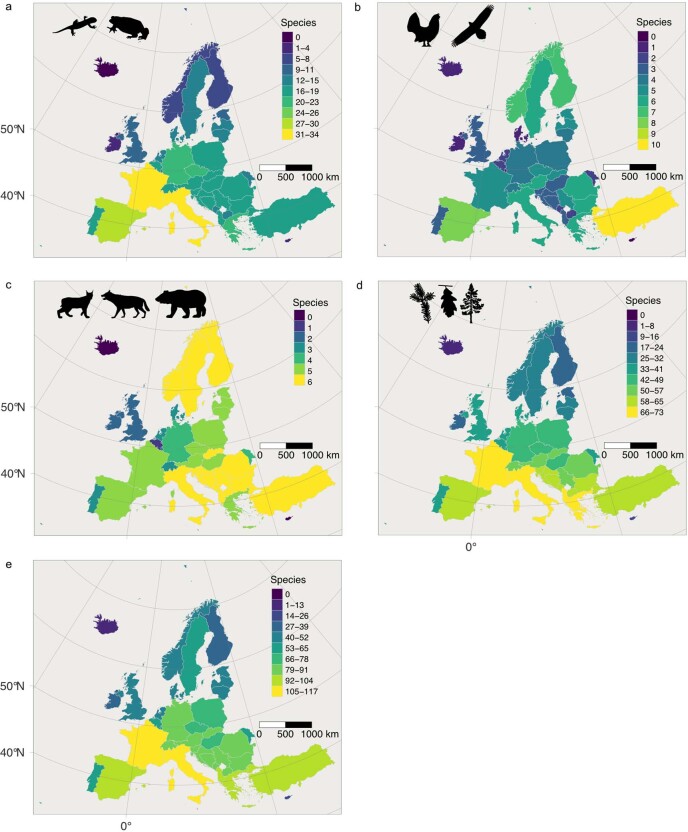
Number of species selected for investigation of patterns of niche marginality in COST Full-Member countries as of 31 January 2021. The number of species with estimated range in each country is shown for (**a)** amphibians, **(b**) a selection of large birds, (**c)** a selection of large carnivores, (**d**) forest trees, and (**e)** all the study species together. The species were chosen for their current or potential future conservation or management interest. While some of the species have been the focus of monitoring of population genetic diversity, most have not, and this was not a requirement for their selection. See Extended Data Table 1 for species identities.

To integrate PGD monitoring into a framework for addressing climate change impacts, we evaluated the relationship between GME and expected climate change effects on species’ trailing-edge climatic niche marginality. These areas correspond to the portion of the least suitable 25% of niche conditions that becomes less suitable with climate change (see the Methods for a full description). The four groups of study species consist of amphibians (44 Anura and 26 Caudata), large birds (16 species in the Accipitridae, Anatidae, Gallidae and Otididae), carnivorans (8 species) and forest trees (91 species), a total of 185 species. The species were chosen for their current or potential future conservation or management interest (Extended Data Table 1) and, except for carnivorans, generally reflect the trend towards greater species richness in southern Europe and the Mediterranean region^32^ (Extended Data Fig. 5). We calculated the values of an index of climate niche marginality^33^ separately for each species and for each pixel within its global range, on the basis of range-wide climate. Pixels with the highest 25% of index values in the species’ range globally indicate the geographic distribution of climatic niche marginality for that species. Current and future distributions of niche marginality for the species in the four study groups are diverse and complex (Appendices 3–6, Supplementary Information). For example, changing spatial patterns of niche marginality and core niche conditions of the Swiss stone pine (*Pinus cembra* L.) produce a geographic mosaic of changing environmental suitability (Fig. 3). The degree of change of niche marginality and the loss of suitable climatic conditions within the species’ current range depend on the severity of predicted climate change, a pattern seen in many other species (Fig. 3b versus Fig. 3c and Fig. 3d versus Fig. 3e; see also Appendices 3–6, Supplementary Information).

**Fig. 4:**
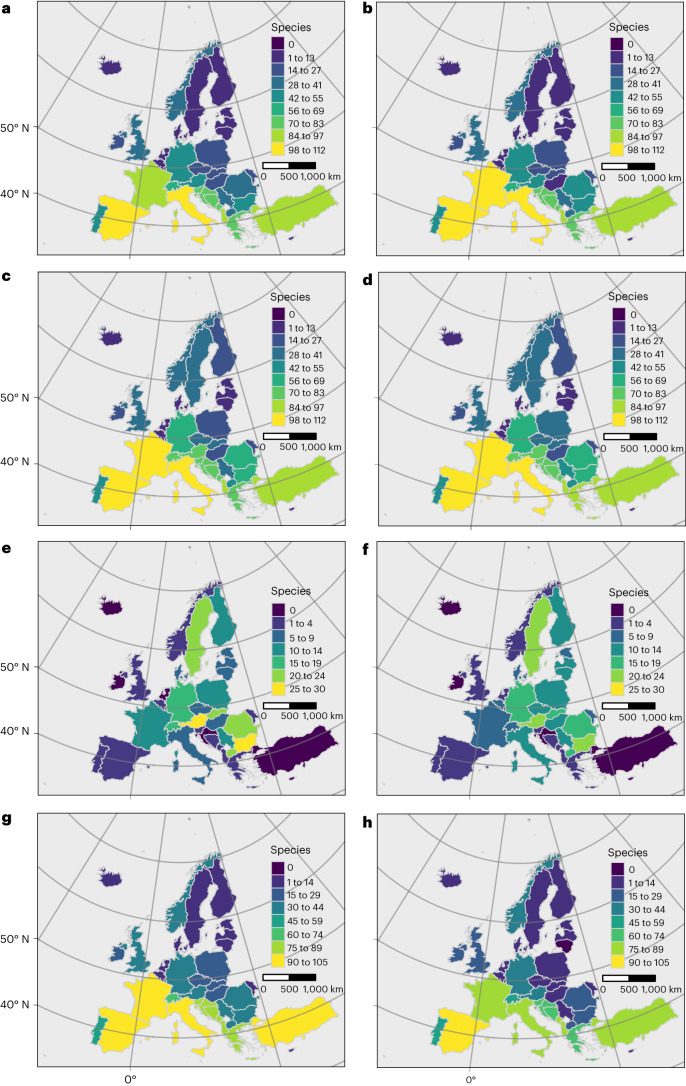
Study species with trailing-edge marginal niche conditions, by country. **a**,**b**, Current trailing-edge marginality. **c**,**d**, Future trailing-edge marginality. **e**,**f**, Species newly experiencing marginal habitat. **g**,**h**, Species experiencing habitat loss. The left panels show the results under harsh climate change (GCM UKESM1-0-LL, SSP 5-8.5); the right panels show the results under mild climate change (GCM MPI-ESM1-2-HR, SSP 3-7.0).

We superimposed the areas of species’ niche marginality at the trailing edge to identify areas where climate change will negatively impact many species. Increases and decreases in the total number of study species with populations at niche margins vary broadly across COST countries but are similar between climate change scenarios (compare Fig. 4a–d). Assuming that species’ climatic niches remain stable over time, we predict increases in the number of species with marginal habitat in Austria, Bulgaria, Germany, Hungary, Poland and Romania. Countries in central and eastern Europe also hold relatively many species that newly experience marginal niche conditions in the future period (Fig. 4e,f). More species lose areas of suitable climatic conditions in southern European countries than elsewhere in Europe (Fig. 4g,h). These trends are similar in other combinations of global circulation model (GCM) and Shared Socioeconomic Pathway (SSP) (Appendix 7, Supplementary Information).

**Extended Data Fig. 6:**
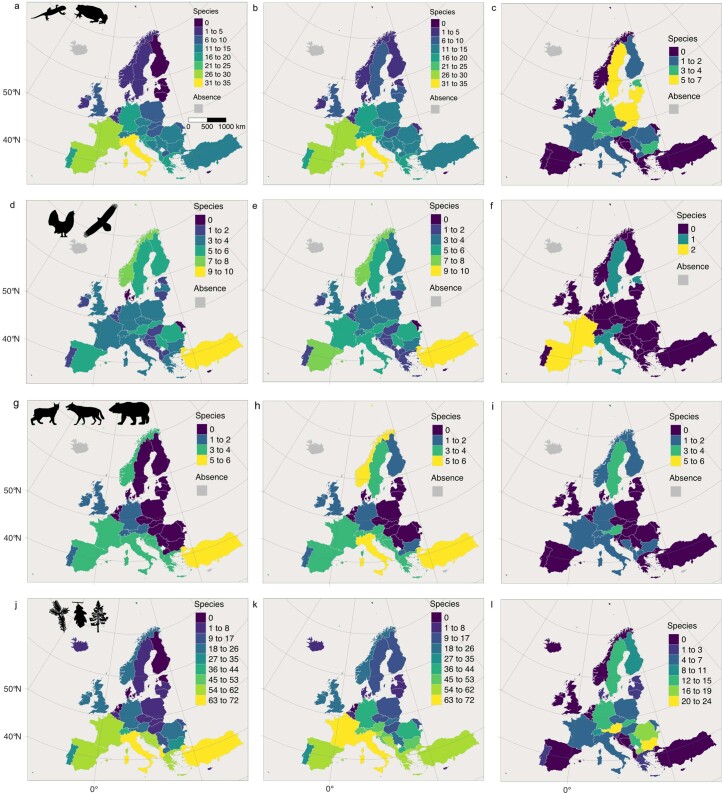
Number of species with trailing-edge, climatic niche margin conditions in each COST country. Data shown for two times, current (1981-2010, left column) and future (2041-2070, center column), and number of species newly with trailing-edge marginal conditions in the future (right column). Values are generated with the UKESM1-0-LL GCM and SSP 5-8.5. The number of species are shown tallied by country for (**a, b, c**) 70 amphibian species, **(d, e, f**) a selection of 16 large bird species, (**g, h, i**) eight large carnivorans, and (**j, k, l**) 91 forest tree species. The patterns of changing joint niche marginality differ among species groups. An increasing number of populations newly at niche margins occurs in central and southeastern Europe in the study amphibians and trees (c, l, respectively). Populations of large carnivorans increasingly experience marginal niche conditions in Austria and Sweden (i). Populations of forest trees increasingly experience marginal niche conditions in southeastern Europe. Countries may show decreasing numbers of species with trailing-edge marginal conditions when populations experience conditions outside their niches in the future, throughout the country (for example, Turkey; j, k). See Appendix S7 in Supplementary Information for analogous data generated with other GCMs and SSPs. See Extended Data Table 1 for species identities.

Spatiotemporal trends in niche marginality in the four groups of species also differ substantially among COST countries (Extended Data Fig. 6 and Appendix 7, Supplementary Information). Across the four groups, the number of species with habitat at niche margins in each country is similar between current and future periods (Extended Data Fig. 6, left column versus middle column). Nonetheless, the number of species of amphibians with climate conditions at niche margins increases in central and eastern Europe (Extended Data Fig. 6a–c), as does the number of large bird species with niche margin conditions, especially in France, Spain and Italy (Extended Data Fig. 6d–f). We predict that the number of carnivorans that experience climates at trailing niche margins will increase in some Nordic countries and in central Europe (Extended Data Fig. 6g–i), providing no evidence of a north–south trend in changing niche marginality in Europe for this taxon. The data suggest that the number of tree species experiencing niche margin conditions will increase broadly across central and northern European countries (Extended Data Fig. 6j–l).

**Fig. 5:**
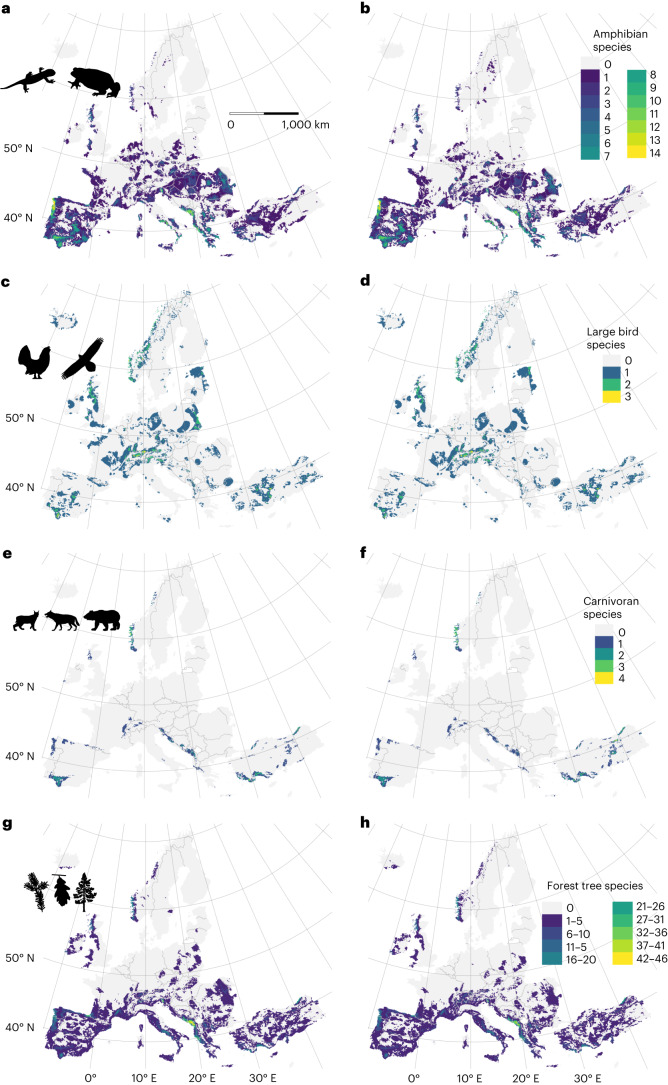
Current species’ joint niche marginality. **a**–**h**, The colours represent the pixel tally of species with trailing-edge niche conditions. The left panels show the results under severe climate change (SSP/GCM: 5-8.5/UKISM1-0-LL). The right panels show the results under milder climate change (3-7.0/MPI-ESM1-2_HR). The data represent 70 amphibian species (**a**,**b**), 16 birds (**c**,**d**), 8 European carnivorans (**e**,**f**) and 91 tree species (**g**,**h**).

**Extended Data Fig. 7:**
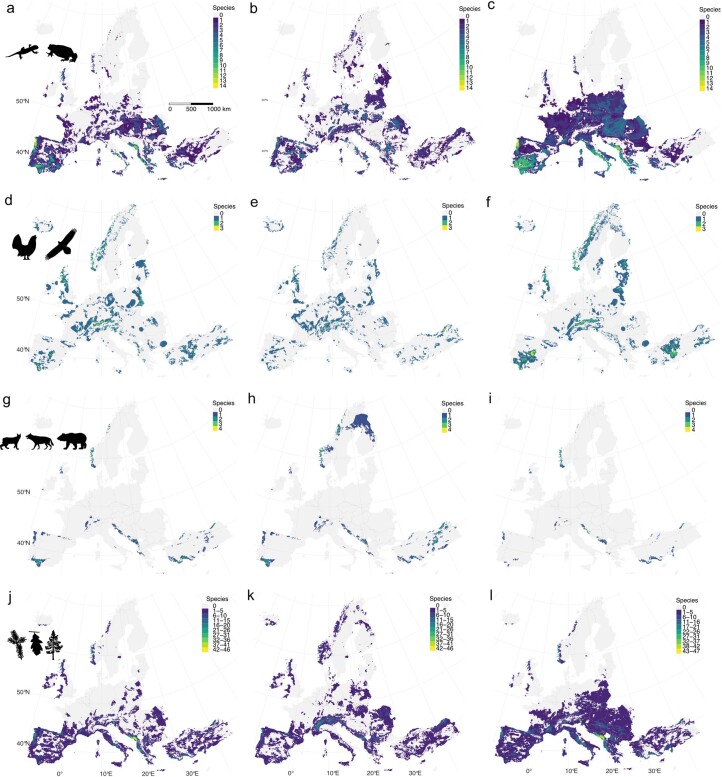
Changes in species joint niche marginality between current (1981-2010) and future (2031–2070). Data are based on the UKESM1-0-LL Global Circulation Model and IPCC Shared Socioeconomic Pathway SSP 5-8.5. Numbers of species in four groups, with (left column) conditions currently within trailing edge niche marginality in COST full member countries; (middle column, dark green and yellow in Fig. 3) number of species with trailing niche marginality in the future time period; (right column, corresponding to red and orange in Fig. 3) number of species losing suitable climatic conditions between the current and future periods. Original data are at the level of 1 km^2^ pixels, but here the data are aggregated to 10 km × 10 km pixels to improve visualization. The highest value within this 100 km^2^ area is displayed. Values are for (**a, b, c**) amphibian species, (**d, e, f**) species of large birds, (**g, h, i**) several large carnivorans, and (**j, k, l**) a collection of forest tree species. Calculation of change in number of species with marginal niche conditions was based on species maps (Appendices S3-6, Supplemental Materials). See Extended Data Table 1 for species identities. See Methods for details on the calculation of climatic niche marginality for species and the filtering of pixels with a CORINE land cover layer.

Regional differences in niche marginality are also visible at the pixel level (Fig. 5 and Extended Data Fig. 7), at which national trends are less apparent. Patterns of current joint niche marginality vary among the four study groups, with foci of joint niche marginality in the Iberian Peninsula and the eastern Adriatic coastline (amphibians and forest trees; Fig. 5a,b,g,h); in the Iberian Peninsula, the Alps and central Turkey (large birds; Fig. 5c,d); and in several restricted areas broadly across Europe (carnivorans; Fig. 5e,f and Appendix 7, Supplementary Information). Current joint niche marginality estimates for different climate scenarios are largely similar (Fig. 5 and Appendix 7, Supplementary Information). The loss of suitable climatic conditions under relatively severe climate change (for example, GCM UKISM1-0-LL/SSP 5-8.5, files with ‘loss’ in the name, Appendix 8, Supplementary Information) leads in the future to areas in which joint niche marginality is reduced (for example, amphibians, Extended Data Fig. 7a–c; trees, Extended Data Fig. 7j–l). Comparison of current and future distributions of niche marginality in individual species often indicates the conversion of core conditions to marginal ones, but not always in the southern portion of species ranges (Appendices 3, 4 and 6, Supplementary Information). Many amphibian and tree species are endemic to Europe, have restricted ranges and lose current areas of suitable conditions with changing climate, including the loss of both niche marginal and core areas. This is analogous to the areas coloured red and orange in Fig. 3 (Extended Data Fig. 7a–c,j–l and Appendices 3, 6 and 8, Supplementary Information). Such losses also occur for some large European birds, especially *Aquila adalberti* (Appendix 4, Supplementary Information). Additional species losing substantial portions of both current trailing-edge marginal and current core niche conditions include *Abies pinsapo*, *Alnus cordata, Alytes cisternasii* and *Lynx pardinus* (Appendices 5 and 6, Supplementary Information). In contrast, species with only a small portion of their range in COST countries, such as wolverine (*Gulo gulo*) and brown bear (*Ursus arctos*), show little change in the distribution of habitat areas having marginal climatic niche conditions in COST countries (Appendix 5, Supplementary Information).

**Fig. 6:**
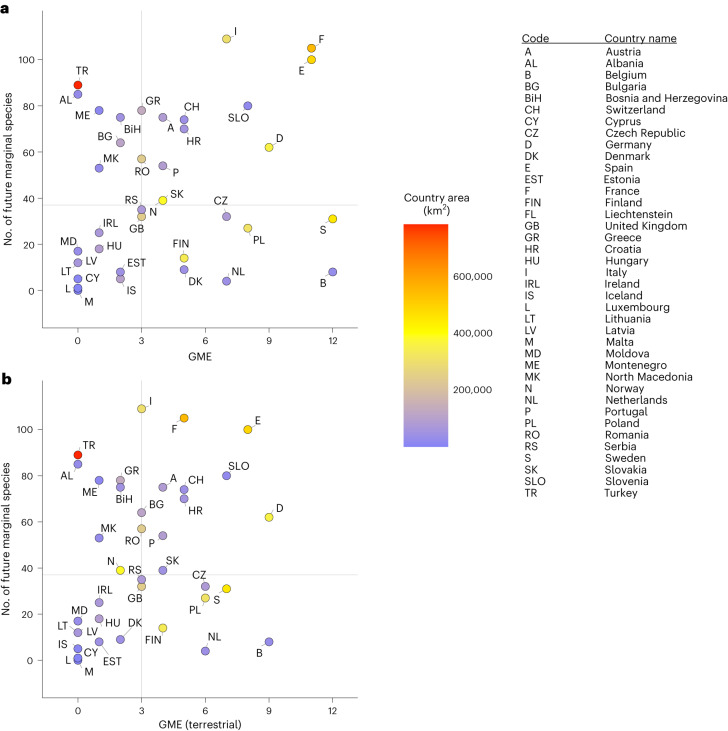
The relationship between GME and the number of species with marginal climatic niche conditions as of the years 2041–2070. **a**, All Category II monitoring as an indicator of effort at the national level. **b**, Programmes to monitor genetic diversity in selected amphibian, avian, carnivoran and plant species only. Countries are indicated by postal codes. Marginal species include all species chosen for the calculation of marginality, including non-troglobite amphibians, a collection of large birds, selected large carnivorans and a set of forest trees. No general linear trends exist, although there is substantial variation both in numbers of species in marginal niche situations and in GME of countries.

**Extended Data Fig. 8:**
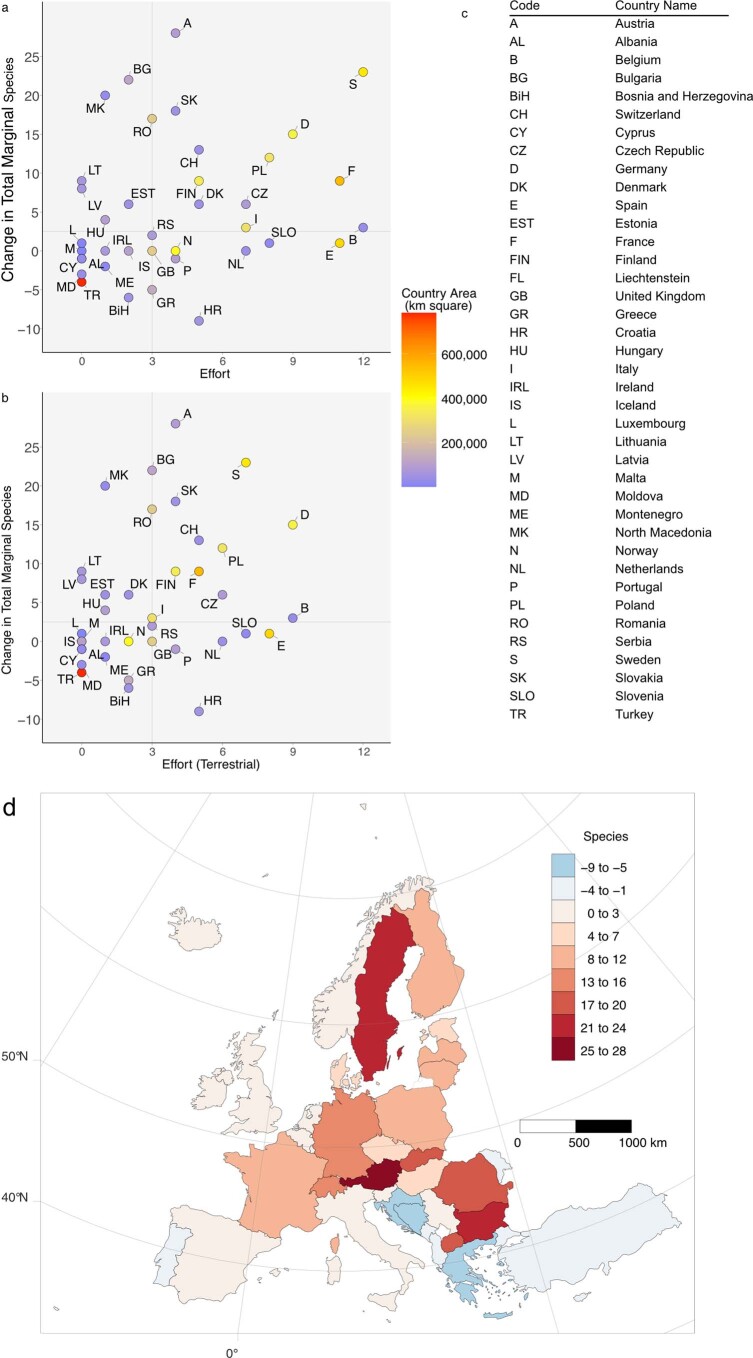
Differences between current and future numbers of species that present trailing-edge niche marginality. Data are based on the UKESM1-0-LL Global Circulation Model and IPCC Shared Socioeconomic Pathway SSP 5-8.5. The panels show (**a**) change in numbers of species with trailing-edge marginal conditions in each country versus monitoring effort, (**b**) change in numbers of trailing-edge marginal conditions versus terrestrial monitoring effort only, (**c**) country codes for ‘a’ and ‘b’, and (**d**) change in total number of species with trailing-edge marginal niche conditions between the current (1981-2010) and future (2031-2070) periods.

The relationship between future joint niche marginality in each country and country GME varies greatly but shows no linear relationship (Fig. 6a). The results are similar when monitoring effort is restricted to terrestrial species only (Fig. 6b), and the order of country values is little affected by choice of GCM and SSP (Appendix 9, Supplementary Information). Countries exhibiting both few study species with trailing-edge niche conditions and relatively little monitoring effort (lower left quadrant in Fig. 6) are of relatively small geographic area, although many smaller countries are not in this quadrant. The results for species’ current marginality versus monitoring effort are similar to these, as are the results for change in the number of marginal species between the two periods versus effort (Appendix 9, Supplementary Information). Variation among countries in the degree of change in the number of species with trailing-edge marginal conditions between the current and future periods is broadly distributed across Europe (Extended Data Fig. 8). The results from other GCM–SSP combinations are similar (Appendix 7, Supplementary Information).

## Discussion

Contrary to our expectations, the areal extent of countries does not generally account for variation in GME. Only by excluding Turkey as an outlier did we observe a positive relationship between country area and GME. Turkey produces population genetic research but is not a member of the European Union (EU). The reporting requirements of the EU Habitats and Birds Directives may successfully promote the use of Category II genetic monitoring. In contrast, and in line with our expectations, countries with relatively low per capita GDP generally have lower GME. However, it appears that countries with intermediate GDP have on average the highest GME. Countries with high GDP are in many cases relatively small (Fig. 2), and many factors conceivably influence the establishment of monitoring programmes, regardless of country size or per capita GDP (such as the number of wild species of traditional or cultural importance, or species richness). Extensive exploration of country characteristics that influence the establishment of programmes for monitoring PGD is beyond the scope of this paper but could be explored in future research, perhaps using data from country reports to the CBD on progress in the implementation of the Kunming-Montreal Global Biodiversity Framework^1,4^.

The monitoring programmes we report here generally focus on detecting changes in population diversity of neutral nuclear marker loci and of mitochondrial DNA (haplotypes). These loci are probably not directly involved in adaptation to climate. The studies minimally report allelic or haplotype diversity, and none are specifically designed to detect genetic responses to climate change or deteriorating environments per se. Genetic characteristics of populations at environmental niche margins could make these populations critical resources for managing the impacts of climate change, such as through translocation programmes^36,37^ (but see ref. ^38^). However, monitoring neutral genetic markers and indicators of effective population size alone is unlikely to provide representative data on the ability of populations to adapt to changing environments (for example, caused by ongoing climate change), because of weak correlations between population genetic marker loci and specific genetic variants affecting functional traits that confer adaptation to the environment^39–41^. Furthermore, the ability of monitoring studies to characterize adaptive potential via measures of genome-wide diversity and/or niche marginality is an ongoing area of research^42–44^. Nonetheless, GME and genetic monitoring using marker loci are suggestive of the future capacity of countries to conduct monitoring of genetic diversity related to predicted or observed climate change responses of species. Countries with large GME should be relatively well prepared to evaluate climate impacts on genetic diversity because they have the relevant infrastructure (that is, genetic laboratories) and experience.

Efforts to increase capacity for genetic monitoring could emphasize southeastern COST countries, where the number of species in areas at climatic niche margins is currently relatively high and expected to remain so in the future (Figs. 4 and 5 and Extended Data Fig. 6). Here, GME for terrestrial species is sparse (Extended Data Fig. 2). These conditions suggest that certain countries (upper left in Fig. 6) present relatively high opportunity/need for climate-guided genetic monitoring and relatively low GME historically. Some commonalities notwithstanding, the areas where species will probably experience environmental deterioration differ depending on the taxonomic group under consideration (Extended Data Fig. 6c,f,i,l). Baseline genetic assessments are needed in some geographic areas, such as the Iberian Peninsula, Italy and France for amphibians and southeastern Europe for forest trees (Extended Data Fig. 6 and Appendices 3 and 6, Supplementary Information), where multiple species will experience environmental deterioration due to rapidly changing climate, as shown by patterns of joint niche marginality (Extended Data Fig. 7c,l). Our results, here based on a joint niche marginality approach, indicate that for various groups of species, the Iberian Peninsula, the eastern Adriatic coast, central Turkey and the Carpathian Mountains can serve as foci for international, cooperative monitoring programmes that anticipate the effects of climate change by establishing genetic baselines that include populations in these areas.

To address the importance of environmental gradients to the conservation of genetic diversity, we distinguish here between populations that are geographically peripheral with regard to a range centroid and populations that are environmentally marginal, occurring towards the limit of their realized environmental niche. Relative geographic position can present little relationship to variation at functional loci, while relative environmental niche marginality of populations can predict variation at these loci and demographic events in populations near niche limits^41,45^. The establishment, adaptation and persistence of populations at environmental niche margins may depend on baseline genetic diversity, the steepness of environmental gradients, the rates of gene flow from non-marginal populations and stochastic processes^20,46^. Monitoring projects should estimate changes in genome-wide diversity when sufficient material and financial resources are available^42^ and should span environmental gradients to include populations from both core and marginal niche situations. Such studies will help elucidate how genetic diversity and adaptive potential vary across species ranges and respond to climate change, something that is not possible without a temporal component in the sampling and analysis^47,48^.

The distributions of some species may be more limited by anthropogenic factors than by climate, such as for some large carnivores. Species’ climatic tolerances may therefore not be well estimated by our methods. For example, the Iberian lynx (*Lynx pardinus*) may lose less area of suitable climatic conditions than estimated here (Appendix 5, Supplementary Information). Examination of these patterns is left for future studies that take focal-species approaches. Furthermore, range expansion with climate change will result in the influx of species into areas with newly suitable climate on leading range edges^16^. In addition, taxonomic revisions (for example, splitting) can change the niche breadth of the revised taxa, with resulting changes in the geographic distribution of areas of niche marginality. Follow-up studies can refine predictions for climatic conditions and niche marginality in the context of taxonomic revisions and specific goals for genetic monitoring and population management. Category II monitoring programmes that span climatic gradients occupied by focal species should be established in additional countries not involved in the COST programme, wherever climate analysis suggests increasing niche marginality of populations of conservation interest, or wherever a risk of genetic erosion is suspected.

Detecting any loss of genetic diversity in niche margin populations should be a priority, and if detected, it should probably trigger a management response. To inform management in this way, monitoring projects need to span entire environmental gradients as occupied by species, in order to sample relevant genetic variation in niche marginal populations. Genetic samples from such prospective monitoring designs will be well suited for evaluating PGD and the adaptive capacity of populations, and for designing appropriate management strategies^49^. Our results may guide future EU investment in genetic monitoring programmes and in conservation genetics/genomics research projects. Positive developments in the support of PGD monitoring that have arisen from the 15th Conference of the Parties to the CBD can be leveraged by adopting language in the EU’s Birds and Habitats Directives to support genetic monitoring. Similar actions should be taken by governments outside of Europe. Future projects may productively focus networking and training efforts more strongly in certain regions, such as the Balkan countries and Turkey.

## Methods

We compared data on GME and climatic niche marginality to address whether historical effort and experience in PGD monitoring at a national scale correspond to the anticipated impacts of climate change on environmental suitability for ensembles of wild species. We call this approach a ‘joint niche marginality’ framework to express how areas of marginal conditions within the niches of multiple species coincide geographically, and we used it to propose taxonomic and geographic foci for future programmes of genetic monitoring. To address our four guiding questions, we report results from a comprehensive survey of the scientific literature, as represented in the Web of Science Core Collection of journals, with use of a simple, inclusive search string of relevant terms. We also collected references and documentation of unpublished monitoring programmes by using professional networks to comprehensively access the grey literature, including governmental and non-governmental reports and web pages in national languages. We focused our analysis exclusively on monitoring programmes that report repeated measures of PGD indicators that were developed with molecular genetic or genomic tools (Category II programmes^34^), and we excluded genetic assessments, which lack temporal replication, from consideration. We compiled and summarized these data by country to address the geographic and taxonomic distribution of monitoring projects as an indicator of GME. We then assembled groups of species of current or potential conservation interest on the basis of taxonomic and functional characteristics and predicted changes in their environmental niche marginality within their current range by using the range-wide occurrence of species, range polygons, and digital land cover and climate layers, the latter of which express current climate and projected changes^33^.

### Distribution of GME in Europe

#### The grey literature

Beginning in October 2019, we began to solicit the submission of published and unpublished (grey literature) materials documenting genetic monitoring programmes, projects and activities (hereafter ‘projects’). We used social media and e-mail to contact the extended network of relationships centred on participants in the COST Action ‘Genomic Biodiversity Knowledge for Resilient Ecosystems’ (G-BiKE, https://www.cost.eu/actions/CA18134/), a Europe-wide effort to improve and promote the use of genetic and genomic methods for supporting the delivery of ecosystem services. We directly contacted colleagues, government officials and non-governmental agency representatives in their home countries to identify and solicit information on past and ongoing projects. Submission of the requested information (Table 1) was open to this broad community of scientists, policymakers and stakeholders and was structured by variables describing each project, organized in an online spreadsheet (Appendix 11, Supplementary Information). We laboured to follow leads and make direct contacts to obtain internal documents and unreleased private reports. We collected all available documentation in the form of web documents and their URLs, white papers, internal and released reports, and published papers that were associated with and substantiated each submitted project. Solicitation and submission of information continued until 31 December 2021. We focused our data collection efforts exclusively on COST full-member countries (hereafter, COST countries) except for those entering COST after the end of data collection: Ukraine, Georgia (31 March 2022) and Armenia (10 November 2022*)*. Submitted projects that did not sample populations in at least one COST full-member country were excluded from subsequent data aggregation and analyses.

**Extended Data Fig. 9:**
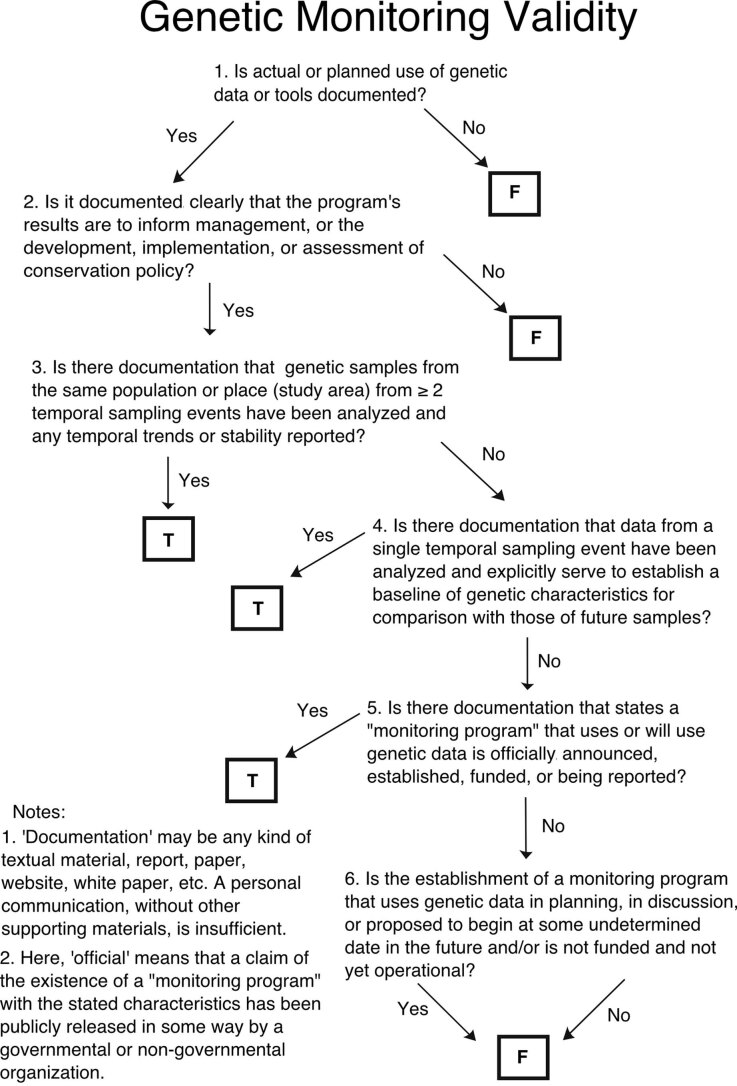
Decision diagram. A flow chart for supporting decisions on the validity of projects as constituting valid Category II genetic monitoring given a wide range of potential documentation, originating in government reports, web documents, and the peer-reviewed literature.

We developed standardized criteria for judging the validity of projects to monitor PGD by following a published definition of genetic monitoring^34^ and by defining a decision tree (Extended Data Fig. 9). Each submitted project was assigned using computer-generated pseudo-random numbers to 2 of 14 evaluators, who sought additional information in national languages as needed through web search and personal inquiries. Pairs of evaluators examined projects independently from one another. When the evaluators disagreed on project validity, they attempted to reach consensus. Persistent disagreements were mediated by two co-authors (P.B.P. and M.B.). Written documentation, broadly defined, was required for a positive decision on project validity, thereby excluding projects reported only by personal communication or e-mail or lacking documentation (Extended Data Fig. 9). Valid monitoring projects included those that acquired and analysed genotype data from the same populations or identical locations, at two or more time points at least one year or one generation apart, whichever was longer. Additionally, candidate projects needed to explicitly declare the goal of informing management and/or conservation policy and activities (Extended Data Fig. 9). These criteria excluded studies lacking temporal replication, studies on pathogens and disease vectors, and studies focused on questions clearly restricted to the field of population biology and without explicit conservation motivation.

A second round of evaluation classified valid monitoring projects into two groups. We distinguished between Category I projects, which collected genotype or haplotype data for species and individual identification, and Category II projects, which reported at least one index of PGD, such as number of alleles, observed or expected heterozygosity, etc.^34^. The use of genetic data from archived samples or collections to establish an initial temporal reference for focal populations was acceptable, as long as the populations were strictly identical. Certain problems were presented by projects that evaluated changes in genetic diversity in reintroduced populations and those receiving introduced individuals to support levels of PGD (that is, genetic support or assisted gene flow)^36^. For the validity of these studies as Category II monitoring, a baseline sample was needed from the population of individuals initially chosen for reintroduction, or repeat temporal samples from the focal, reintroduced or supported population itself. We excluded projects comparing genetic diversity in contemporary samples to that from the original or putative source populations when these were sampled only after (re)introductions, due to the potential for sampling bias. As in the initial evaluation of validity, both evaluators needed to express a consensus concerning the type of monitoring (Category I or II) that was conducted.

#### The scientific literature

We also conducted a separate survey of the peer-reviewed scientific literature to identify projects monitoring genetic diversity. On 1 December 2021, one co-author (P.B.P.) conducted a search of all Web of Science collections with the search string “Topic: ‘genetic population diversity monitoring’ NOT ‘cell’ NOT ‘virus’ NOT ‘medical’”. The citations were then filtered to come only from the following journal categories: Agriculture, Agronomy, Dairy Animal Science, Biodiversity Conservation, Marine Freshwater Biology, Ecology, Entomology, Environmental Sciences, Evolutionary Biology, Fisheries, Forestry, Genetics and Heredity, Horticulture, Multidisciplinary, Multidisciplinary Sciences, Ornithology, Plant Sciences and Zoology. Other strategies, such as additionally restricting the search to COST countries, resulted in the omission of studies that qualified as Category II monitoring in Europe. One co-author (P.B.P.) scored all collected citations for being conducted in COST countries and for being either Category I or II monitoring. Each of these candidate studies was re-examined independently by one of four additional co-authors (D.R., E.B., A.K. and F.E.Z.) to evaluate the initial assessment and to identify redundancy within the original list of validated projects. Confirmed, non-redundant cases were then added to the list of monitoring projects. Ad hoc repetition of the Web of Science search to identify additional studies published in late 2021 and efforts to obtain documentation of specific unpublished projects, produced before the end of 2021, continued during the first four months of 2022.

We focused on Category II monitoring studies because of their relevance to mandates to conserve genetic diversity, and we carefully tallied these studies by country and by taxonomic and additional groupings (Appendix 11, Supplementary Information). We considered submitted projects that monitored particular single species in a country as distinct projects when different populations were studied by different research groups, institutes or organizations. We also considered projects conducted by a single research group but having more than one focal species as distinct. Projects addressing different focal populations of a single species, analysed as exclusive, distinct sets of populations by a single research group, were also counted as distinct projects. Nonetheless, publications that presented analyses of repeated samples from a single set of populations, and were extensions of original studies and used the original published data in establishing temporal trajectories of genetic diversity, were not counted as separate projects regardless of author identity. Analyses of samples by contract laboratories, in a separate country from that of the study population(s), research group or monitoring organization, did not qualify the project to count towards the tally of projects for that separate country, unless of course at least one sampled population came from that country. In multi-country projects generally, samples for genetic analysis needed to be physically collected within a country for a project to count towards the tally of projects in that country. This meant that potentially a project was assigned (tallied) only to a subset of participating countries, those that were the sources of genetic samples. Projects reporting a temporal trajectory of genetic diversity in captive or domestic populations needed to employ genetic analysis of repeated samples and not rely exclusively on estimates of genetic diversity or change thereof that were obtained from pedigree analysis of breeding records. Because some projects sampled populations in more than one country, we defined the GME of a country as the tally of Category II monitoring projects obtaining genetic data from within the country. We determined the geographic distribution of GME for focal taxonomic and functional species groups by mapping GME for each group in each COST country and examining the frequency distribution of GME among countries. We focus our analyses exclusively on Category II monitoring studies and will address Category I studies in a future publication.

### Climate niche marginality in Europe

#### Focal species

We defined four divergent groups of species for the examination of current and future geographical patterns of climatic conditions. Our objective was to construct groups with membership that exceeded the scope of current GME and that, because of taxon identity or life history traits, either are currently of conservation interest or could conceivably become of interest as climate change proceeds. Thus, while many of the species may be on national Red Lists in European countries, this was not a requirement for inclusion. We also did not attempt to comprehensively include species of conservation interest. We explicitly disregarded membership on Red Lists and EU Directives as criteria because the varying completeness, taxonomic resolution and criteria for species’ inclusion of national Red Lists across Europe made it impossible to implement a single standard. Furthermore, not all COST countries are members of the EU and subject to the Directives. We developed lists of focal taxa to include (1) most native European Amphibia (44 Anura and 26 Caudata), because of their recognized sensitivity to climate change (we excluded cave-dwelling amphibians because of their limited exposure to terrestrial climate); (2) 16 species of large birds, representing the Accipitridae, Anatidae, Gallidae and Otididae, because size is related to extinction probability in birds globally^50^; (3) a set of 8 relatively large carnivorans because of their general economic, ecological and cultural importance; and (4) a set of 91 species of forest trees (64 Magnoliopsida and 27 Pinopsida), because of the general economic and cultural importance of trees (Extended Data Table 1). We focused on these groups because the range maps for the species are probably reliable, and the occurrence data are probably well reported. Global range maps for each focal species were retrieved as polygons from the data portal of the International Union for the Conservation of Nature (IUCN)^51^, and species occurrence data were retrieved from the Global Biodiversity Information Facility^52–55^. We then defined species’ global distributions as the pixels occupied by the species according to the IUCN range maps. We refined species’ distributions within range polygons by filtering out pixels corresponding to CORINE Land Cover 2018 habitat classes^56^ that were not intersected at least once by occurrences of the species in question. This removed urban areas and other habitat/land-cover types for which we found no evidence of occupation in the occurrence data.

#### Marginality calculations

We used the worldwide 19 bioclimatic variables from the Chelsa database of global climate values for the period 1981–2010 at 30 arcsec resolution (http://chelsa-climate.org^57^) to calibrate principal component scores. We defined a working environmental space consisting of the first two principal component axes. This space summarized the main climatic gradients present on Earth (75.7% of the variation explained). We rasterized the IUCN species range maps at 30 arcsec resolution, extracted bioclimatic values for every occupied pixel (after filtering with CORINE 2018) and projected these values to the global climate space to generate species scores^58^. Using these scores, we delineated the niche margins of each species by kernel density estimation (that is, the 0.99 quantile)^33,58^. These margins described the boundaries of the climatic conditions currently occupied by the species globally. Finally, we calculated the Niche Margin Index (NMI), a standardized metric of climate marginality, for each pixel of each species distribution, on the basis of the multivariate distance to the niche margins and using the approach of Broennimann et al.^33^. The NMI metric for each species varies from 0 to 1, with values of 0 indicating that the climatic conditions in the pixel are at the niche margin and values of 1 indicating conditions at the niche centre. To provide synthetic niche marginality maps for each species, we considered that pixels with the 25% most marginal conditions for a species (NMI < 0.25), determined globally, constituted climatically marginal areas for the species, while the rest of the pixels within the species’ niche constituted the core conditions. In this way, species’ niche marginality scores translated directly from a multivariate space to a geographic distribution within the current range of the species (for example, Fig. 3). Notably, niche marginal situations can occur in both geographically central and peripheral areas within the species range.

To map the future distribution of marginality of species’ climate niches, we updated the climatic values of pixels corresponding to the species distributions within the study area using two SSP scenarios, the relatively moderate SSP 3-7.0 (regional rivalry) and the worst-case SSP 5-8.5 (fossil fuel development). We chose these scenarios because growth in carbon emissions currently is not showing evidence of moderation^59^. We chose three GCMs, IPSL-CM6A-LR, UKISM1-0-LL and MPI-ESM1-2_HR, to incorporate substantial variability among GCM models in the analysis. This provided six combinations of SSP scenarios and GCMs. We then obtained the simulated climate data for a baseline period, 1981–2010, and for a 30-year future period, 2041–2070, from the Chelsa database v.2.1 (ref. ^60^). We recalculated the NMI metric for each species in each pixel to produce maps of species’ future niche marginality and the transition of areas between core and marginal niche categories for each SSP–GCM combination. We identified leading and trailing niche marginal areas by examining how NMI values changed over the time interval: NMI increases for leading edge pixels (conditions move closer to the niche centroid), while trailing edge pixels exhibit decreasing NMI values (conditions move further from the niche centroid). The number and distribution of trailing-edge pixels and changes in NMI values can vary among the SSP–GCM combinations. Mapping the geographic distribution of future niche marginality in this way assumes that the climate niches of species fulfil the assumptions of niche stability^61,62^ and niche filling^63^. Multispecies marginality maps were produced for each species group by stacking the species’ marginality maps and calculating maps of the number of species in marginal niche conditions for each pixel, at present and in the future. We compared maps of current and future niche marginality to identify pixels in which we estimated that populations of species will shift into climatically marginal niche conditions in the future, and other changes determined by changing NMI values.

To facilitate the comparison of GME to the predicted effects of climate change on species’ niche situations at the country level, we converted species maps of niche marginality to country tallies of species with trailing marginal niche conditions and tallied change over time. For each COST country, we obtained a shapefile of country boundaries at 10 m resolution from the Natural Earth website^64^. We excluded overseas territories and regions of European countries—that is, islands and areas outside of a rectangular bounding box defined by 25° W, 57° E, 29.1° N and 73° N. This excluded, for example, the Canary Islands (Spain), Svalbard (Norway) and French Guiana (France). We used the R package tmap^65^ to map the number of PGD monitoring projects in each country, the number of marginal species in each focal taxonomic group currently and in the future, the predicted number of species that newly experience niche margin conditions within a country and the count of species that lose suitable niche conditions. We plotted counts of species, using current and future joint niche marginality, and their change between periods against country tallies of PGD monitoring programmes. These plots allow visualization of the relationship between national effort for PGD monitoring and geographic foci of future climatic niche margin conditions for multiple species.

### Statistical analyses

We compared GME among countries by modelling the number of Category II monitoring projects as a function of two national indicators. We used country area as an example indicator of the physical aspects of countries, and we estimated the land area of COST countries in continental Europe, in the Mediterranean and Baltic islands, and in Asia using the R package sf^66^. While many more physical aspects could be explored, a comprehensive study of these aspects of COST countries is beyond the scope of the paper. We also chose per capita GDP as an example indicator of economic activity and available resources, one that is available for all COST countries. Data on GDP in 2020 US dollars were obtained from an authoritative online source^67^, the most recent year for which data from all COST countries were available. The relationship of monitoring effort with many other social and economic indicators could be explored, but we leave this as well for future analyses. On the basis of inspection of scatter plots, country area entered the models as a first-order effect, while GDP entered as a second-order orthogonal polynomial.

We used a generalized linear model (GLM) framework to analyse country counts of PGD monitoring projects. Models were fitted with functions from the R packages stats, MASS and hermite^68,69^. Outlier and influential data points were identified with leverage statistics and by inspection. We quantified model explanatory capacity with the Veall–Zimmermann^70^ pseudo-*R*^2^ calculated on deviance residuals, and we used model likelihoods and *χ*^2^ statistics to compare models during model development. We modelled the data with Poisson, negative binomial and Hermite regressions and based statistical decisions on negative binomial models because of a significant reduction in overdispersion of residuals in comparison with the Poisson model, and no additional improvement provided by the Hermite model (Appendix 2, Supplementary Information). We examined negative-binomial GLM residuals for small-scale spatial autocorrelation (SAC) using a randomization test of the significance of Moran’s *I* (H_0_: *I* = 0), at successive intervals of 300 km between country centroids, using the correlog function in the R package ncf^71^. We did not address large-scale spatial structure (>1,500 km). Because SAC can bias tests of significance of model effects when analysing spatial data, we removed SAC from GLM residuals by first constructing spatial eigenvectors (Moran’s Eigenvector Maps) with the function mem from the R package adespatial^72^ and a regional distance network among country centroids constructed with the functions dnearneigh and nb2listw in the R package spdep^73^ (Appendix 2, Supplementary Information). Eigenvectors with positive eigenvalues were included as additional linear terms (regardless of statistical significance) in the GLMs. We added eigenvectors until the *P* values of the randomization test of Moran’s *I*, calculated on model residuals at intervals to 1,500 km, equalled or exceeded 0.05 after rounding. Although significance levels were reduced by the addition of spatial eigenvectors, decisions concerning the statistical significance of model terms were not affected. All tests of significance were two-tailed. Mapping and statistics were conducted in R version 4.1.2 (ref. ^74^).

### Reporting summary

Further information on research design is available in the Nature Portfolio Reporting Summary linked to this article.

### Supplementary information

Reporting Summary

Peer Review File

### Source data

Source Data Fig. 1 Country-specific data on the number of monitoring projects for species groups.

Source Data Fig. 2 Country data on area and per capita GDP.

## Figures and Tables

**Extended Data Table 1:**
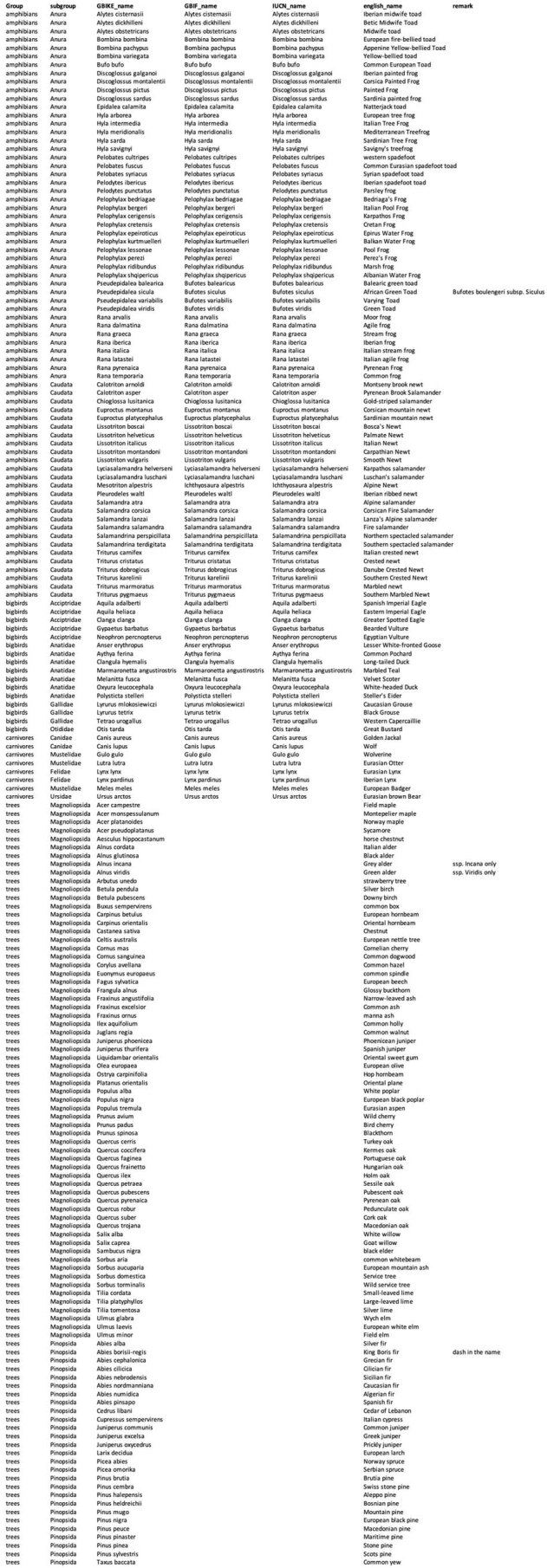
Species of current or potential future conservation or management interest and used in this study for examining niche marginality

## Data Availability

The raw data on submitted candidate monitoring projects and a variable indicating their validity as Category II monitoring are available as Appendix S11 and for download at https://figshare.com/s/296e3bf1db7b84ec71bd. All data used in the study are available in compressed archives in a Zenodo repository^75^ at 10.5281/zenodo.8417583. Source data are provided with this paper.

## Code availability

The code to produce all graphics and statistical analyses, along with the necessary raw and intermediate data, can be found in compressed archives in a Zenodo repository^75^ at 10.5281/zenodo.8417583.

## References

[1] McOwen CJ (2016). Sufficiency and suitability of global biodiversity indicators for monitoring progress to 2020 targets. Conserv. Lett..

[2] Laikre L (2020). Post-2020 goals overlook genetic diversity. Science.

[3] Hoban S (2023). Genetic diversity goals and targets have improved, but remain insufficient for clear implementation of the post-2020 global biodiversity framework. Conserv. Genet..

[4] *Decision Adopted by the Conference of the Parties to the Convention on Biological Diversity: Kunming-Montreal Global Biodiversity Framework* Decision XV/4 (Convention on Biological Diversity, 2022).

[5] Ette J-S, Geburek T (2021). Why European biodiversity reporting is not reliable. Ambio.

[6] O’Brien D (2022). Bringing together approaches to reporting on within species genetic diversity. J. Appl. Ecol..

[7] Hoban S (2014). Comparative evaluation of potential indicators and temporal sampling protocols for monitoring genetic erosion. Evol. Appl..

[8] Hoban S (2020). Genetic diversity targets and indicators in the CBD post-2020 Global Biodiversity Framework must be improved. Biol. Conserv..

[9] Pereira HM (2013). Essential biodiversity variables. Science.

[10] *Monitoring Framework for the Kunming-Montreal Global Biodiversity Framework* Decision XV/5 (Convention on Biological Diversity, 2022).

[11] Leigh DM, Hendry AP, Vazquez-Dominguez E, Friesen VL (2019). Estimated six per cent loss of genetic variation in wild populations since the industrial revolution. Evol. Appl..

[12] Miraldo A (2016). An Anthropocene map of genetic diversity. Science.

[13] Exposito-Alonso M (2022). Genetic diversity loss in the Anthropocene. Science.

[14] Merilä J, Hendry AP (2014). Climate change, adaptation, and phenotypic plasticity: the problem and the evidence. Evol. Appl..

[15] Purvis, A. M. et al. in *Global Assessment Report of the Intergovernmental Science-Policy Platform on Biodiversity and Ecosystem Services* (eds Brondízio, E. S. et al.) Ch. 2.2 (IPBES Secretariat, 2019).

[16] Hampe A, Petit RJ (2005). Conserving biodiversity under climate change: the rear edge matters. Ecol. Lett..

[17] Carvalho SB, Torres J, Tarroso P, Velo-Anton G (2019). Genes on the edge: a framework to detect genetic diversity imperiled by climate change. Glob. Change Biol..

[18] Razgour O (2019). Considering adaptive genetic variation in climate change vulnerability assessment reduces species range loss projections. Proc. Natl Acad. Sci. USA.

[19] Bridle JR, Vines TH (2007). Limits to evolution at range margins: when and why does adaptation fail?. Trends Ecol. Evol..

[20] Kawecki TJ (2008). Adaptation to marginal habitats. Annu. Rev. Ecol. Evol. Syst..

[21] Rehfeldt GE, Ying CC, Spittlehouse DL, Hamilton DA (1999). Genetic responses to climate in *Pinus contorta*: niche breadth, climate change, and reforestation. Ecol. Monogr..

[22] Bontrager M (2021). Adaptation across geographic ranges is consistent with strong selection in marginal climates and legacies of range expansion. Evolution.

[23] Carnaval AC, Hickerson MJ, Haddad CFB, Rodrigues MT, Moritz C (2009). Stability predicts genetic diversity in the Brazilian Atlantic Forest hotspot. Science.

[24] Hewitt GM (2004). Genetic consequences of climatic oscillations in the Quaternary. Phil. Trans. R. Soc. Lond. B.

[25] Nadeau CP, Urban MC (2019). Eco-evolution on the edge during climate change. Ecography.

[26] Aitken SN, Yeaman S, Holliday JA, Wang TL, Curtis-McLane S (2008). Adaptation, migration or extirpation: climate change outcomes for tree populations. Evol. Appl..

[27] Holliday JA, Suren H, Aitken SN (2012). Divergent selection and heterogeneous migration rates across the range of Sitka spruce (*Picea sitchensis*). Proc. R. Soc. B.

[28] Wessely J (2022). Climate warming may increase the frequency of cold-adapted haplotypes in alpine plants. Nat. Clim. Change.

[29] Flanagan SP, Forester BR, Latch EK, Aitken SN, Hoban S (2018). Guidelines for planning genomic assessment and monitoring of locally adaptive variation to inform species conservation. Evol. Appl..

[30] *COST Members* (COST Association, 2023); https://www.cost.eu/about/members/

[31] Cervellini M (2021). Diversity of European habitat types is correlated with geography more than climate and human pressure. Ecol. Evol..

[32] Myers N, Mittermeier RA, Mittermeier CG, da Fonseca GAB, Kent J (2000). Biodiversity hotspots for conservation priorities. Nature.

[33] Broennimann O (2021). Distance to native climatic niche margins explains establishment success of alien mammals. Nat. Commun..

[34] Schwartz MK, Luikart G, Waples RS (2007). Genetic monitoring as a promising tool for conservation and management. Trends Ecol. Evol..

[35] G-BiKE Working Group 2. Monitoring project submissions. *Figshare* https://figshare.com/s/296e3bf1db7b84ec71bd (2023).

[36] Aitken SN, Whitlock MC (2013). Assisted gene flow to facilitate local adaptation to climate change. Annu. Rev. Ecol. Evol. Syst..

[37] Hoegh-Guldberg O (2008). Assisted colonization and rapid climate change. Science.

[38] Van Daele F, Honnay O, De Kort H (2022). Genomic analyses point to a low evolutionary potential of prospective source populations for assisted migration in a forest herb. Evol. Appl..

[39] Bonin A, Nicole F, Pompanon F, Miaud C, Taberlet P (2007). Population adaptive index: a new method to help measure intraspecific genetic diversity and prioritize populations for conservation. Conserv. Biol..

[40] Willi Y, Van Buskirk J, Hoffmann AA (2006). Limits to the adaptive potential of small populations. Annu. Rev. Ecol. Evol. Syst..

[41] Dauphin B (2020). Disentangling the effects of geographic peripherality and habitat suitability on neutral and adaptive genetic variation in Swiss stone pine. Mol. Ecol..

[42] Kardos M (2021). The crucial role of genome-wide genetic variation in conservation. Proc. Natl Acad. Sci. USA.

[43] Reed DH, Frankham R (2001). How closely correlated are molecular and quantitative measures of genetic variation? A meta-analysis. Evolution.

[44] Harrisson KA, Pavlova A, Telonis-Scott M, Sunnucks P (2014). Using genomics to characterize evolutionary potential for conservation of wild populations. Evol. Appl..

[45] Perez-Navarro MA (2022). Comparing climatic suitability and niche distances to explain populations responses to extreme climatic events. Ecography.

[46] Bridle JR, Polechova J, Kawata M, Butlin RK (2010). Why is adaptation prevented at ecological margins? New insights from individual-based simulations. Ecol. Lett..

[47] Clark, R. D. et al. The practice and promise of temporal genomics for measuring evolutionary responses to global change. *Mol. Ecol. Resour.* **00**, 1–17 (2023); 10.1111/1755-0998.13789 10.1111/1755-0998.13789 PMC12142728 36961384

[48] Jensen, E. L. & Leigh, D. M. Using temporal genomics to understand contemporary climate change responses in wildlife. *Ecol. Evol.* **12**, e9340 (2022). 10.1002/ece3.9340 PMC9481866 36177124

[49] Thurman LL (2020). Persist in place or shift in space? Evaluating the adaptive capacity of species to climate change. Front. Ecol. Environ..

[50] Hughes EC, Edwards DP, Thomas GH (2022). The homogenization of avian morphological and phylogenetic diversity under the global extinction crisis. Curr. Biol..

[51] *The IUCN Red List of Threatened Species: Spatial Data* (International Union for the Conservation of Nature, 2022); https://www.iucnredlist.org/resources/spatial-data-download

[52] *GBIF Occurrence Download, Large Carnivorans* (Global Biodiversity Information Facility, 2021); 10.15468/dl.8skxjd

[53] *GBIF Occurrence Download, Forest Trees* (Global Biodiversity Information Facility, 2021); 10.15468/dl.guf53c

[54] *GBIF Occurrence Download, Amphibians* (Global Biodiversity Information Facility, 2021); 10.15468/dl.z88kj8

[55] *GBIF Occurrence Download, Large Birds* (Global Biodiversity Information Facility, 2021); 10.15468/dl.zyhtqq

[56] *Copernicus Land Monitoring Service 2018* (European Environment Agency, European Union, 2018); https://land.copernicus.eu/pan-european/corine-land-cover/clc2018

[57] Karger DN (2017). Climatologies at high resolution for the Earth land surface areas. Sci. Data.

[58] Broennimann O (2012). Measuring ecological niche overlap from occurrence and spatial environmental data. Glob. Ecol. Biogeogr..

[59] Liu Z, Deng Z, Davis S, Ciais P (2023). Monitoring global carbon emissions in 2022. Nat. Rev. Earth Environ..

[60] Karger DN (2017). Datadescriptor: climatologies at high resolution for the Earth’s land surface areas. Sci. Data.

[61] Guisan A, Petitpierre B, Broennimann O, Daehler C, Kueffer C (2014). Unifying niche shift studies: insights from biological invasions. Trends Ecol. Evol..

[62] Pearman PB, Guisan A, Broennimann O, Randin CF (2008). Niche dynamics in space and time. Trends Ecol. Evol..

[63] Svenning JC, Skov F (2004). Limited filling of the potential range in European tree species. Ecol. Lett..

[64] *Large Scale Data, 1:10m* (Natural Earth, 2021); https://www.naturalearthdata.com/

[65] Tennekes M (2018). tmap: thematic maps in R. J. Stat. Softw..

[66] Pebesma E (2018). Simple features for R: standardized support for spatial vector data. R J..

[67] *DataBank: World Development Indicators* (World Bank Group, 2022); https://data.worldbank.org/indicator/NY.GDP.PCAP.CD

[68] Moriña D, Higueras M, Puig P, Oliveira M (2018). hermite: generalized Hermite distribution. R package version 1.1.2. R J..

[69] Venables, W. N. & Ripley, B. D. *Modern Applied Statistics with S* 4th edn (Springer, 2002).

[70] Veall MR, Zimmermann KF (1994). Evaluating pseudo-*R*^2^s for binary probit models. Qual. Quant..

[71] Bjornstad, O. N. ncf: Spatial covariance functions. R package version 1.3-2 (2022).

[72] Dray, S. et al. adespatial: Multivariate multiscale spatial analysis. R package version 0.3-16 (2022).

[73] Bivand, R. S., Pebesma, E. & Gomez-Rubio, V. *Applied Spatial Data Analysis with R* 2nd edn (Springer, 2013).

[74] R Core Team. R: A Language and Environment for Statistical Computing v.4.1.2 (R Foundation for Statistical Computing, 2021).

[75] Pearman, P. B. & Broennimann, O. Data, code, and supplementary materials for Pearman P. B., Broennimann, O., et al. Monitoring species genetic diversity in Europe varies greatly and overlooks potential climate change impacts. *Zenodo*10.5281/zenodo.8417583 (2023).

