# Supplementary material for: Monitoring of species’ genetic diversity in Europe varies greatly and overlooks potential climate change impacts
Source: Nat Ecol Evol. 2024 Jan 15;8(2):267–81. doi: 10.1038/s41559-023-02260-0 (PMC10857941; doi:10.1038/s41559-023-02260-0)
Supplement: Supplementary file 1 — Reporting Summary [file 41559_2023_2260_MOESM1_ESM.pdf]

nature portfolio

Corresponding author(s): Peter B. Pearman

Last updated by author(s): Aug 1, 2023

Reporting Summary

Nature Portfolio wishes to improve the reproducibility of the work that we publish. This form provides structure for consistency and transparency in reporting. For further information on Nature Portfolio policies, see our Editorial Policies and the Editorial Policy Checklist.

Statistics

For all statistical analyses, confirm that the following items are present in the figure legend, table legend, main text, or Methods section.

n/a	Confirmed	
☐	☒	The exact sample size (n) for each experimental group/condition, given as a discrete number and unit of measurement
☒	☐	A statement on whether measurements were taken from distinct samples or whether the same sample was measured repeatedly
☐	☒	The statistical test(s) used AND whether they are one- or two-sided<br>*Only common tests should be described solely by name; describe more complex techniques in the Methods section.*
☐	☒	A description of all covariates tested
☐	☒	A description of any assumptions or corrections, such as tests of normality and adjustment for multiple comparisons
☐	☒	A full description of the statistical parameters including central tendency (e.g. means) or other basic estimates (e.g. regression coefficient) AND variation (e.g. standard deviation) or associated estimates of uncertainty (e.g. confidence intervals)
☐	☒	For null hypothesis testing, the test statistic (e.g. F, t, r) with confidence intervals, effect sizes, degrees of freedom and P value noted<br>*Give P values as exact values whenever suitable.*
☒	☐	For Bayesian analysis, information on the choice of priors and Markov chain Monte Carlo settings
☒	☐	For hierarchical and complex designs, identification of the appropriate level for tests and full reporting of outcomes
☒	☐	Estimates of effect sizes (e.g. Cohen's d, Pearson's r), indicating how they were calculated

Our web collection on statistics for biologists contains articles on many of the points above.

Software and code

Policy information about availability of computer code

Data collection	Not complicated. Only a Google sheet was used to collect candidate monitoring cases. Climate data, species range polygons, and occurrence data were acquired from public sources.
Data analysis	Analyses are described in detail in the text. Methods of spatial statistics and control of spatial autocorrelation are described in the on-line methods section. Statistical code and results are provided in Appendix S2. Raw data and code for marginality calculations and mapping is provided in a Zenodo repository.

For manuscripts utilizing custom algorithms or software that are central to the research but not yet described in published literature, software must be made available to editors and reviewers. We strongly encourage code deposition in a community repository (e.g. GitHub). See the Nature Portfolio guidelines for submitting code & software for further information.

Data

Policy information about availability of data

All manuscripts must include a data availability statement. This statement should provide the following information, where applicable:

- Accession codes, unique identifiers, or web links for publicly available datasets
- A description of any restrictions on data availability
- For clinical datasets or third party data, please ensure that the statement adheres to our policy

Original candidate monitoring project submissions are included in Appendix S11 and a DOI for the data set at a repository is provided in a data availability

statement.

Research involving human participants, their data, or biological material

Policy information about studies with human participants or human data. See also policy information about sex, gender (identity/presentation), and sexual orientation and race, ethnicity and racism.

Reporting on sex and gender	n/a
Reporting on race, ethnicity, or other socially relevant groupings	n/a
Population characteristics	n/a
Recruitment	n/a
Ethics oversight	n/a

Note that full information on the approval of the study protocol must also be provided in the manuscript.

Field-specific reporting

Please select the one below that is the best fit for your research. If you are not sure, read the appropriate sections before making your selection.

☐ Life sciences ☐ Behavioural & social sciences ☒ Ecological, evolutionary & environmental sciences

For a reference copy of the document with all sections, see nature.com/documents/nr-reporting-summary-flat.pdf

Ecological, evolutionary & environmental sciences study design

All studies must disclose on these points even when the disclosure is negative.

Study description	This study addresses monitoring of population genetic diversity and the detection of potential genetic effects of climate change. We collected comprehensive information on monitoring projects for population genetic diversity (PGD), for purposes of management and conservation, in EU-COST program full-participant countries. The monitoring projects were tallied by various functional and taxonomic species groups, and by country. The number of PGD monitoring projects in each country was used as an indicator of genetic monitoring effort (GME) for PGD monitoring. We modeled GME as a function of fundamental economic and geographical descriptors to identify factors associated with differences in monitoring activity among countries. We also modeled and mapped core and marginal conditions of the species climate niches (climate niche marginality), under current and potential future climatic conditions, for four groups of species of current and/or potential future conservation interest: Amphibians, and selections of large birds, carnivorans, and forest trees. We produced maps of these distributions in COST countries, and additionally mapped two derived variables: (i) the increases in the number of species newly experiencing marginal niche conditions, (ii) numbers of study species experiencing any loss of area with suitable climate conditions, as indicators of environmental degradation associated with predicted climate change. These values were combined across species to map the changing distribution of species joint niche marginality in COST countries that is associated with ongoing climate change. Country values of current and predicted future joint niche marginality were plotted against country GME to infer how countries vary in need for monitoring and effort (and potentially capacity) to monitor, identify, and manage the genetic impacts of ongoing climate change on populations of conservation interest.
Research sample	We collected comprehensive data on projects monitoring population genetic diversity for conservation and management purposes in COST-member countries, constituting a census of such projects across all COST full-member countries, as of the start of data collection. These data were gathered using professional and stakeholder networks, and through a structured search of the Web of Science. We also acquired range and occurrence data on selected species in four target groups: European amphibians, and selected large birds, carnivorans, and forest trees. These species were selected to comprise a set of species that represents current and potential future conservation and management interest across all COST full-member countries, regardless of EU membership. Range maps originated from the IUCN and occurrence data from the Global Biodiversity Information Facility. We also acquired climate data for the current period and for a future period spanning the years 2041-2070 from the Chelsa database vers. 2.1. These were chosen in order to estimate current and future climate niche marginality for the target species. Finally, we used landcover/landuse data from the CORINE 2018 layer in order to filter the IUCN range maps for inappropriate habitat types, thereby refining our estimate of species current distributions.
Sampling strategy	We made every effort to census genetic monitoring projects, constructing a comprehensive data set. Target species were selected to represent taxonomically divergent species of known or potential future conservation and management interest.
Data collection	Data on candidate projects for evaluation as Category II projects that monitor population genetic diversity over time, as defined by a peer-reviewed, published source, were collected through professional networks centered on population geneticists and practitioners involved in the COST Action 'Genomic Biodiversity Knowledge for Resilient Ecosystems (G-BiKE, https://www.cost.eu/actions/CA18134/)'. This was done to discover monitoring efforts that had not necessarily appeared in the peer-reviewed literature. Additionally, a search of the Web of Science was conducted using broad search terms and a selection of relevant journals.

Timing and spatial scale	Data were collected between 10.2019 and 31.21.2021. Data from the Web of Science were collected during 12.2021. Additional information was collected during 1.1.2022-30.4.2022 to identify substantiating documentation dated prior to 1.1.2022.
Data exclusions	Candidate monitoring projects and those gathered through search of the Web of Science were evaluated for validity as Category II monitoring projects, following Schwarz et. al 2007. TREE 22:25-33. Candidate projects not passing this validation and projects without samples from at least one COST full-member country were excluded.
Reproducibility	Validity of submitted, candidate projects for monitoring population genetic diversity was evaluated independently by at least two persons. When consensus was not reached, the first and/or last author served as a tie-breaking opinion.
Randomization	Each submitted candidate project was evaluated for validity (following Schwarz et al 2007) by randomly assigning the project to two evaluators who worked independently, then attempted to reach a consensus opinion.
Blinding	none

Did the study involve field work? ☐ Yes ☒ No

Reporting for specific materials, systems and methods

We require information from authors about some types of materials, experimental systems and methods used in many studies. Here, indicate whether each material, system or method listed is relevant to your study. If you are not sure if a list item applies to your research, read the appropriate section before selecting a response.

Materials & experimental systems

n/a	Involved in the study	
☒	☐	Antibodies
☒	☐	Eukaryotic cell lines
☒	☐	Palaeontology and archaeology
☒	☐	Animals and other organisms
☒	☐	Clinical data
☒	☐	Dual use research of concern
☒	☐	Plants

Methods

n/a	Involved in the study	
☒	☐	ChIP-seq
☒	☐	Flow cytometry
☒	☐	MRI-based neuroimaging
